# Genome-wide association analysis and functional annotation of positional candidate genes for feed conversion efficiency and growth rate in pigs

**DOI:** 10.1371/journal.pone.0173482

**Published:** 2017-06-12

**Authors:** Justyna Horodyska, Ruth M. Hamill, Patrick F. Varley, Henry Reyer, Klaus Wimmers

**Affiliations:** 1 Teagasc, Food Research Centre, Ashtown, Dublin, Ireland; 2 Leibniz Institute for Farm Animal Biology (FBN), Institute for Genome Biology, Dummerstorf, Germany; 3 Hermitage Genetics, Kilkenny, Ireland; 4 Faculty of Agricultural and Environmental Sciences, University Rostock, Rostock, Germany; Universita degli Studi di Bologna, ITALY

## Abstract

Feed conversion efficiency is a measure of how well an animal converts feed into live weight and it is typically expressed as feed conversion ratio (FCR). FCR and related traits like growth rate (e.g. days to 110 kg—D110) are of high interest for animal breeders, farmers and society due to implications on animal performance, feeding costs and environmental sustainability. The objective of this study was to identify genomic regions associated with FCR and D110 in pigs. A total of 952 terminal line boars, showing an individual variation in FCR, were genotyped using 60K SNP-Chips. Markers were tested for associations with estimated breeding values (EBV) for FCR and D110. For FCR, the largest number of associated SNPs was located on chromosomes 4 (30 SNPs), 1 (25 SNPs), X (15 SNPs) and 6 (12 SNPs). The most prominent genomic regions for D110 were identified on chromosomes 15 (10 SNPs), 1 and 4 (both 9 SNPs). The most significantly associated SNPs for FCR and D110 mapped 129.8 Kb from *METTL11B* (chromosome 4) and 32Kb from *MBD5* (chromosome 15), respectively. A list of positional genes, closest to significantly associated SNPs, was used to identify enriched pathways and biological functions related to the QTL for both traits. A number of candidate genes were significantly overrepresented in pathways of immune cell trafficking, lymphoid tissue structure, organ morphology, endocrine system function, lipid metabolism, and energy production. After resequencing the coding region of selected positional and functional candidate genes, six SNPs were genotyped in a subset of boars. SNPs in *PRKDC*, *SELL*, *NR2E1* and *AKRIC3* showed significant associations with EBVs for FCR/D110. The study revealed a number of chromosomal regions and candidate genes affecting FCR/D110 and pointed to corresponding biological pathways related to lipid metabolism, olfactory reception, and also immunological status.

## Background

Improving feed conversion efficiency (FCE) in pigs is a major goal in pig breeding as feed accounts for a high proportion of the total production cost [1]. Selection for improved FCE is also a key factor in reducing the environmental footprint of the pig industry [2]. FCE can be defined as a measure of an animal’s efficiency in converting feed into live weight [3] and it is typically expressed as feed conversion ratio (FCR, ratio of feed intake to weight gain) [4]. As such, growth rate traits which determine the weight gain in different developmental stages are closely related to FCR thus having a direct impact on efficiency [5]. However, phenotypic and genetic correlations between FCR and its components (i.e. feed intake and body weight gain) have been reported to be higher for FCR and feed intake compared to FCR and body weight gain in different pig populations [5]. Apart from FCR, other indexes such as residual feed intake (RFI), which can be described as the difference between an individual’s actual feed intake and its predicted feed requirements for maintenance and growth, have been studied [2].

A number of quantitative trait loci (QTLs) affecting feed efficiency in pigs have been detected (PigQTLdb, http://www.animalgenome.org/cgi-bin/QTLdb/SS/index). However, most of them were identified using a linkage mapping approach resulting in wide genomic QTL regions. Such linkage data is limited to within-family selection only [6]. A genome-wide association study (GWAS) approach would offer the potential for improved accuracy and refinement in the identification of QTL locations at the population level [7]. To date, only a few studies have used the GWAS approach to identify QTLs for FCE traits in pigs. Sahana et al. [6] detected a number of significant QTLs for FCR on porcine chromosomes (SSC) 4, 7, 8 and 14 in Duroc pigs. Another study identified only one QTL for FCR on SSC 4 in Duroc boars [8]. A GWAS performed on Yorkshire boars revealed several QTLs influencing RFI on SSC 7 and 14 [9]. Do et al. [10] additionally reported QTLs on SSC 3, 8, 9, 10, 15 and 17 for RFI in Yorkshire pigs. The same author also conducted a study on Duroc boars and identified significant regions for RFI on SSC 1, 8, 9, 13 and 18 [11]. While employing the GWAS approach, the objective of this study was to identify genomic regions associated with feed efficiency in an important commercial pig sire line (Maxgro, Hermitage Genetics).

## Materials and methods

### Animals and phenotypes

Animal care, slaughter and tissue collection of the animals used in this study were performed in compliance with national regulations related to animal research and commercial slaughtering and were approved by the local committees for the care and use of animals of the Teagasc Research Center Ashtown and the Leibniz Institute for Farm Animal Biology. A total of 952 Maxgro boars, which is predominately Pietrain based terminal line, were used in this study. These animals, born between year 2006 and 2012, were selected as replacement boars in the artificial insemination (AI) stud and were supplied by Hermitage Genetics (Ireland). The pigs were penned in groups of fourteen with a space allowance of 0.75 m^2^ per pig and were fed a pelleted finisher diet (National Research Council, 2012) consisting of 177.8 g crude protein, 5.0 g tP, 6.0 g Ca and 13.9 MJ DE, and 8.8 g ileal digestible lysine per kilogram. They also had *ad libitum* access to water through nipple drinkers. Phenotypic data such as FCR and D110 comprising 46 and 91 percent of the total number of animals used in the study, respectively, were recorded by Hermitage Genetics following the method of Varley et al [12]. Breeding values (EBV) for FCR (range: -0.44–0.32, mean: -0.09, SD: 0.09) and D110 (range: -20.8–9.18, mean: -10.2, SD: 4.00) were estimated using Best Linear Unbiased Prediction (BLUP) system [13] from a dataset that included multiple breeds, two sexes and a number of farms and AI studs. The models for the routine estimation of direct genetic effects for both traits were multivariate and included fixed effects of contemporary group, pig breed and sex. The affiliation of a pig to a litter was fit as an uncorrelated random effect in the prediction. Moreover, the status of performance testing was also included as fixed effect. Specifically, for performance tested pigs individual feeding records were obtained using a single-space computerised feeder (Mastleistungsprüfung MLP-RAP; Schauer Agrotronic AG, Sursee, Switzerland) [12]. The pigs (age at start of the test—mean: 102.3 days, SD: 6.4 days; age at end of the test—mean: 144.1 days, SD: 6.4 days) were weighted at the start (mean: 60.9 kg, SD: 7.6 kg) and the end (mean: 109.3 kg, SD: 9.9 kg) of the test period for a minimum of 40 days (mean: 41.8 days, SD: 4.7 days). Based on these observations FCR was calculated. In order to obtain the number of days needed to gain a final body weight of 110 kg, the pig’s date of birth and slaughter weight, which is slightly above or below 110 kg, was entered into the BLUP system and calculated. For the prediction of the EBVs FCR, both start weight and end weight were fit as a covariate in order to consider weight related differences in feed efficiency. Following the test period, boars were entered into the AI stud therefore no euthanasia of boars occurred.

### SNP array genotyping, quality control and statistical analysis

Approximately 50 ml of blood from *Vena jugularis* was collected from each boar by Hermitage Genetics into a tube containing EDTA. Genomic DNA was extracted from the preserved blood using QIAamp DNA Blood Mini Kit (QIAGEN Ltd., West Sussex, UK) according to manufacturer’s instructions. Genotyping with PorcineSNP60 BeadChip (Illumina Inc., San Diego, CA, USA) was performed in compliance with the SNP Infinium HD assay protocol (http://www.illumina.com). Subsequently, data was analysed using GenomeStudio (Version 2011.1, Illumina Inc.). Individuals with call rate ≤ 97% and SNPs with call frequency ≤ 95% and minor allele frequency (MAF) ≤ 0.03 were excluded. The departure from Hardy-Weinberg equilibrium (HWE) was not considered as indicator for consistent genotyping errors as it has been reported to be underpowered for this purpose [14].

After quality control, remaining SNPs were tested for an association with EBVs FCR and D110. SNP-trait association analysis was implemented with a mixed linear model using JMP Genomics 6 software (version 6, SAS INST., Inc., Cary, NC, 2002–2010). In order to correct for population structure, the relationship matrix tool implemented in JMP Genomics 6 was used to compute identity-by-state (IBS) relations between individuals based on genotype data [15]. After compression of K matrices, these relations were included as a random effect in the model. Moreover, this factor accounting for relatedness was applied to counteract high false-positive rates and the misestimating of QTL effects assigned to the usage of EBVs for GWAS [16]. Threshold p-values for suggestive and Bonferroni-adjusted genome-wide significance were set to -log10[p-value] = 4.7 (1 divided by 48440 independent tests) and -log10[p-value] = 6 (0.05 divided by 48440 independent tests), respectively.

A list of genes closest to the significant SNPs (-log_10_[p-value] ≥ 6) was created allowing a maximum distance of 1Mb between the marker and genes, using the Ensembl database (http://www.ensembl.org, release 78) and was uploaded into Ingenuity Pathways Analysis (IPA; Ingenuity^®^ Systems, http://www.ingenuity.com) in order to investigate relevant pathways and functional categories. Benjamini-Hochberg corrected P values were used to map the genes to the most significant molecular, cellular and physiological systems development functions (P < 0.01). To get insights into the most relevant metabolic and signalling pathways based on the designated list of genes, canonical pathways were displayed (Fisher’s exact test; P < 0.05), although they did not differ significantly after Benjamini-Hochberg correction. Categories addressing human disease and disorder-associated pathways were excluded from the IPA analysis.

### Validation of candidate genes

Twelve genes with functions relevant to feed efficiency according to IPA were selected from the candidate gene list for validation and further analysis. A set of primers for each gene was designed based on published sequence data (Ensembl database) using Primer3 (http://primer3.ut.ee/) (Table 1). Genomic DNA of low EBV FCR pigs (n = 10, mean: -0.182, SD: 0.027) and high EBV FCR pigs (n = 10, mean: 0.040, SD: 0.032), with a p-value of difference < 0.0001, was pooled (n = 2) and used as template for PCR. All PCR reactions were carried out in a final volume of 50 μl and consisted of 10 μl PCR buffer (5x) (Promega, WI, USA), 3 μl MgCl_2_ (25mM) (Promega), 0.4 μl dNTP mix (10mM each), 0.4 μl of each primer (100 pmol, Eurofins MWG Operon, Germany), 0.4 μl Go Taq DNA Polymerase (100U, Promega), 30 ng of the DNA pool and filled with dH_2_O. The cycling conditions were as follows: initial denaturation at 95°C for 135 sec; 35 cycles of 95°C for 45 sec, annealing for 45 sec (60°C for *OPRD1*, *WDTC1*, *SMPD2* and 56°C for the remaining primers), and 72°C for 75 sec, subsequently final extension of 72°C for 10 min. PCR products were subjected to electrophoresis on 1.5% agarose gels and visualised. PCR products were purified using the QIAquick PCR Purification Kit (QIAGEN Ltd.) and sequenced commercially (Eurofins, MWG-Biotech). Chromatograms were analysed to identify segregating SNP.

**Table 1:** Forward and reverse primers for PCR amplification of the twelve selected positional candidate genes located within 1Mb of the genome-wide significant markers for EBVs FCR and D110.

Gene	Ensembl reference	Size (bp)	Forward	Reverse
*CD164*	*ENSSSCG00000004414*	713	TGTGTCTGTCCAGTTTCTTCGC	TGAAGTCAGGCTGGGGATTACG
*NR2E1*	*ENSSSCG00000004384*	706	TCTCCCTTCCCTCTCTTCACCT	ACCTACGCTGCCCTCTGATTTC
*SMPD2*	*ENSSSCG00000004408*	697	CCTCCTCTCTGACCCTCTCTCT	TGGGGCTGTCTGTTTCTTCC
*PRKDC*	*ENSSSCG00000006274*	735	AGGAAACACGCCTCAGTTGGTA	ACGCAGGAGACAGAAGGAAAGC
*SELL*	*ENSSSCG00000006287*	706	TCTCAAAACAAATGTCTGTGGCTGT	GGTTATCTTCTGGGCAACTCACC
*SELP*	*ENSSSCG00000006288*	350	ACCTGAATCCAACCTCTCTCCA	TGCATCTGAAGTAGCAAGTCGT
*OPRD1*	*ENSSSCG00000027401*	718	GCTCCCATCCACATCTTCGTCA	CCCCTCAATTCCACCTTCCTCA
*WDTC1*	*ENSSSCG00000003570*	567	CCAGGGACCAAGACAACCGA	CACCATACCTCACAGCAACGC
*AKR1C3*	*ENSSSCG00000030447*	792	GCTGACACTTAGCAGTTGAGGAATA	GGTGGAGGAAAGAGGAGTTAAATACA
*KLF6*	*ENSSSCG00000028828*	702	GACCAACAGCCTGAACTCGGA	CCCTGAGTCTCACTTCCCCAAA
*MBD5*	*ENSSSCG00000015667*	773	ACTTGGAAGCCCTGATGTTTTCAC	ACCCTATCGTTGACCTTGGTGAC
*MMADHC*	*ENSSSCG00000028646*	696	GGATTCTCCGTTGATGATCTTGGC	CCTTATTCTTCTTTCCCGCACAAAC

Based on the PCR product sequencing of the twelve genes, six confirmed SNPs (located in *MBD5*, *OPRD1*, *AKR1C3*, *NR2E1*, *PRKDC* and *SELL*) were selected for genotyping in 436 Maxgo boars as a representative subset of animals. The SNP genotyping was performed using TaqMan^®^ SNP Genotyping Assays (Applied Biosystems, Foster City, CA, USA). Each 15 μl PCR reaction consisted of 7.5 μl of TaqMan^®^ genotyping master mix (Applied Biosystems, Foster City, CA, USA), 0.375 μl 40 x genotyping assay mix (Applied Biosystems), 6.125 μl dH_2_O and 1 μl of genomic DNA (10 ng/μl). Thermal cycling was performed using ABI PRISM^®^ 7500 Real Time PCR System (Applied Biosystems) and the cycling conditions were as follows: initial denaturation at 95°C for 10 min, followed by 40 cycles of 95°C for 15 sec denaturation and 60°C for 1 min annealing/extension. Genotype calling was carried out using proprietary 7500 System SDS software (Applied Biosystems).

Allele frequencies were computed and deviations from HWE (p-value < 0.05) were tested using Haploview software [17]. Mixed linear model using JMP Genomics 6 software (version 6, SAS INST., Inc., Cary, NC, 2002–2010) was used to evaluate associations between the four SNPs with allele frequency as predicted and greater than 5% (rs340456509, rs80900450, rs319738340 and rs81508945) and EBVs for FCR and D110 in the Maxgro boars (n = 436). Compressed IBS relations were included as a random effect in the model. In order to determine additive and dominant effects for the particular SNP, indicator variables alpha (1 = homozygote for the allele with higher least square means, -1 = homozygote for the allele with lower least square means and 0 = heterozygote), and delta (1 = heterozygote and 0 = homozygote) were created. Regression models were performed, using EBV FCR and EBV D110 as the dependent variables and variable alpha and delta as the independent variables, to estimate the additive and dominant effects for each significant SNP (REG procedure of the SAS v9.3 software package). Based on the squared multiple correlation (R^2^) of the regression, the effect size was expressed as the phenotypic variance attributable to the genetic variance at the designated locus.

## Results

### Genome-wide association study

**Fig 1:**
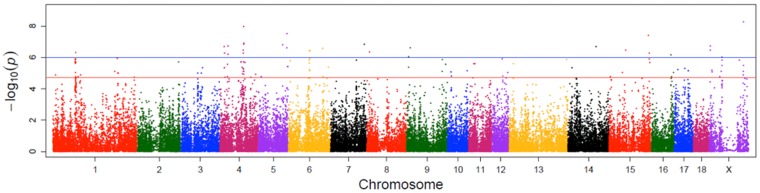
Manhattan plot of the genome-wide association analysis of EBV FCR. The red and blue lines indicate the suggestive (-log10[p-value] ≥ 4.7) and the Bonferroni-adjusted genome-wide significance threshold (-log10[p-value] ≥ 6), respectively.

After quality control, 940 individuals and 48,440 SNPs, mapped to the Sscrofa 10.2 pig genome assembly, remained for the further analysis. In total 132 SNPs reached the threshold of suggestive significance for an association with EBV FCR (-log_10_[p-value] ≥ 4.7) (Fig 1; S1 Table). The largest number of associated SNPs were located on SSC4 (30 SNPs) and SSC1 (25 SNPs) followed by SSCX (15 SNPs) and SSC6 (12 SNPs). A total of 25 SNPs mapping to 10 porcine autosomes crossed the Bonferroni-adjusted genome-wide significance threshold (-log_10_[p-value] ≥ 6). Of the 25 SNPs, 5 were located within a 2.37 Mb segment on SSC4 and pointed to Methyltransferase like 11B (*METTL11B*) and Coagulation Factor V (*F5*) as positional candidate genes (Table 2). A search for genes in the window surrounding the significantly associated markers revealed Selectin L (*SELL*), Selectin P (*SELP*) and Protein kinase, DNA-activated, catalytic polypeptide (*PRKDC*) as putative candidate genes for FCE. On SSC15, Neuronal guanine nucleotide exchange factor (*NGEF*) and 5-hydroxytryptamine (serotonin) receptor 2B, G protein-coupled (*HTR2B*) genes were revealed as functional candidate genes, whereas *DIS3* and *ARL4C* were identified as positional candidates. Two significant SNPs on SSC6 were located near Feline Gardner-Rasheed sarcoma viral oncogene homolog (*FGR*) and Protein tyrosine phosphatase, receptor type, U (*PTPRU*). A further search for genes with putative relevance for processes related to FCE in this region revealed Tetratricopeptide repeats 1 (*WDTC1*) and Opioid receptor, delta 1 (*OPRD1*). Furthermore, a significant SNP mapped to SSC1 was located in an uncharacterised gene and the nearest annotated gene was CD164 molecule, sialomucin (*CD164*). Nuclear receptor subfamily 2, group E, member 1 (*NR2E1*) and Sphingomyelin phosphodiesterase 2, neutral membrane (*SMPD2*) were identified as functional candidate genes in this region.

**Table 2:** Genes located closest to the genome-wide significant SNPs.

EBV^$^	SNP	Neglog_10_ (p-value)	SSC*	Position (bp)	Region	Nearest gene*	Gene position (bp)
FCR	H3GA0002102	6.32	1	84,686,166	Intronic	*ENSSSCG00000004415*	84,644,862–84,709,541
FCR	MARC0000845	6.26	4	86,747,415	Intergenic	*ENSSSCG00000024309*	86,796,081–86,804,148
FCR	ALGA0026204	6.47	4	87,021,547	Intergenic	*MCM4*	87,134,012–87,185,073
FCR	H3GA0013204	7.96	4	88,311,790	Intergenic	*METTL11B*	88,441,595–88,460,670
FCR	ALGA0026230	6.84	4	89,104,182	Intronic	*F5*	89,027,936–89,109,573
FCR	ALGA0026233	6.91	4	89,118,147	Intergenic	*F5*	89,027,936–89,109,573
FCR	ASGA0028724	6.44	6	78,297,229	Intergenic	*FGR*	78,358,088–78,326,491
FCR	ALGA0035847	6.43	6	80,577,487	Intergenic	*PTPRU*	80,106,273–80,024,322
FCR	MARC0015113	7.40	15	146,404,317	Intronic	*DIS3L2*	146,381,891–146,596,424
FCR	ALGA0119312	6.27	15	149,350,761	Intergenic	*ARL4C*	149,122,784–149,123,362
D110	ALGA0060013	6.32	10	72,375,760	Intergenic	*AKR1C3*	72,091,036–72,106,952
D110	H3GA0030777	6.92	10	72,766,001	Intergenic	*KLF6*	72,992,245–73,001,823
D110	MARC0036947	6.92	15	2,640,639	Intergenic	*LYPD6B*	2,443,675–2,456,793
D110	ALGA0115976	6.89	15	2,798,633	Intronic	*KIF5C*	2,730,359–2,901,565
D110	ALGA0113899	6.78	15	2,835,746	Intronic	*KIF5C*	2,730,359–2,901,565
D110	MARC0072361	7.75	15	2,843,921	Intronic	*KIF5C*	2,730,359–2,901,565
D110	ALGA0083417	8.15	15	3,322,649	Intergenic	*MBD5*	3,354,689–3,361,520

^$^Estimated breeding value;

*Sscrofa 10.2 assembly

**Fig 2:**
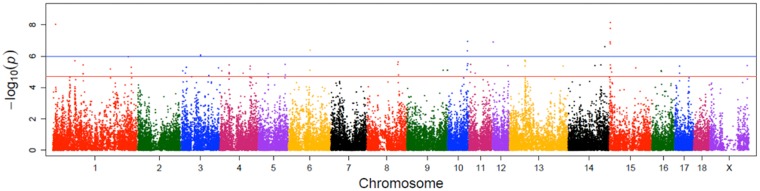
Manhattan plot of the genome-wide association analysis of EBV D110. The red and blue lines indicate the suggestive (-log10[p-value] ≥ 4.7) and the Bonferroni-adjusted genome-wide significance threshold (-log10[p-value] ≥ 6), respectively.

In total 71 SNPs reached the threshold of suggestive significance for an association with EBV D110 (-log_10_[p-value] ≥ 4.7) (Fig 2; S1 Table). The largest number of associated SNPs was located on SSC15 (10 SNPs), SSC1 and SSC4 (9 SNPs), SSC3 (8 SNPs), followed by SSC10 and 13 (5 SNPs). A total of 12 SNPs mapping to 7 porcine autosomes crossed the Bonferroni-adjusted genome-wide significance threshold (-log_10_[p-value] ≥ 6). Of the 12 SNPs, 5 were located within a 682 Kb segment (between 2.64 and 3.32 Mb) on SSC15. Three of these markers were located within an intron of Kinesin family member 5C (*KIF5C*) gene (Table 2). Two remaining markers mapped near Methyl-CpG binding domain protein 5 (*MBD5*) and LY6/PLAUR domain containing 6B (*LYPD6B*). A further search for genes revealed Methylmalonic aciduria cblD type, with homocystinuria (*MMADHC*) with functional relations to D110. On SSC10, Kruppel-like factor 6 (*KLF6*) and Aldo-keto reductase family 1, member C3 (*AKR1C3*) were identified as positional candidate genes with a putative contribution to D110.

### Functional enrichment among mapped genes

A total of 86 and 16 genes mapped within 1Mb upstream and downstream of significant markers for EBV FCR and D110, respectively, were uploaded into Ingenuity Pathways Analysis. Functional annotation of the positional candidate genes to biological processes and canonical pathways (top 5) is presented in Tables 3 and 4. The top canonical pathways significantly overrepresented among the positional candidate genes for EBV of FCR were related to cell cycle control, estrogen receptor signaling, RXR and subfamily 1 nuclear receptors activation, granulocyte mediate inflammation, and sphingomyelin metabolism. Functional annotation revealed organismal development and organ morphology, lymphoid tissue and hematological system development, and immune cell trafficking to be significantly enriched among the genes located in QTL regions for EBV FCR. Moreover, bile acid and androgen biosynthesis, TR/RXR Activation, methylglyoxal detoxification, and retinoate biosynthesis pathways were the top pathways associated with the positional candidate genes for EBV D110. Furthermore, statistically associated biological functions with the positional candidate genes for EBV D110 were energy, lipid and drug metabolism, endocrine system development, and small molecule biochemistry.

**Table 3:** Top molecular themes for the positional and functional candidate genes located within 1Mb of the genome-wide significant markers for EBV of FCR and D110.

EBV	Category	B-H p-value*	Genes
FCR	Hematological System Development and Function	8.53E-05-9.68E-02	*CEBPD*, *SRSF4*, ***SELL***, *ATPIF1*, *FGR*, *SELE*, *F5*, *THEMIS2*, *EPB41*, *HTR2B*, ***SELP***, *ZBTB24*, *WASF2*, ***PRKDC***, *FOXO3*, ***OPRD1***
FCR	Immune Cell Trafficking	8.53E-05-9E-02	***SELP***, ***SELL***, *FGR*, *SELE*, *F5*, *FOXO3*, ***OPRD1***
FCR	Lymphoid Tissue Structure and Development	2.89E-03-9E-02	*THEMIS2*, ***SELP***, ***SELL***, *SELE*, *F5*, ***PRKDC***
FCR	Organ Morphology	2.89E-03-9.68E-02	*CEBPD*, ***SELL***, *ECEL1*, ***NR2E1***, ***SELE***, *CHRNG*, *WASF1*, *GPR3*, *EIF4E2*, *HTR2B*, *CHRND*, ***SELP***, *KIFAP3*, *SEC63*, *SYTL1*, ***PRKDC***, *FOXO3*
FCR	Organismal Development	2.89E-03-9.14E-02	*CEBPD*, ***SELL***, *ATPIF1*, ***NR2E1***, *FGR*, *SELE*, *F5*, *NPPC*, *WASF1*, *GPR3*, *THEMIS2*, *EIF4E2*, *HTR2B*, ***SELP***, *KIFAP3*, *WASF2*, ***PRKDC***, *FOXO3*
D110	Endocrine System Development and Function	4.7E-06-8.62E-02	***AKR1C3***, ***MBD5***, *ACVR2A*, *AKR1C1/AKR1C2*, *AKR1C4*
D110	Small Molecule Biochemistry	4.7E-06-6.21E-02	*PFKP*, ***AKR1C3***, ***MBD5***, *ACVR2A*, *AKR1C1/AKR1C2*, *AKR1C4*
D110	Energy Production	3.51E-05-2.45E-02	***AKR1C3***, *AKR1C1/AKR1C2*, *AKR1C4*
D110	Lipid Metabolism	6.06E-05-5.44E-02	***AKR1C3***, *AKR1C1/AKR1C2*, *AKR1C4*
D110	Drug Metabolism	3.68E-04-3.01E-02	***AKR1C3***, *AKR1C1/AKR1C2*, *AKR1C4*

*Range of B-H multiple testing correction p-values of enriched biological functions within the category; candidate genes selected for downstream validation are highlighted in bold

**Table 4:** Top canonical pathways for the positional and functional candidate genes located within 1Mb of the genome-wide significant markers for EBV of FCR and D110.

EBV	Ingenuity Canonical Pathways	P-value	Genes
FCR	Cell Cycle Control of Chromosomal Replication	4.66E-03	*RPA2*, *MCM4*
FCR	Estrogen Receptor Signaling	1.27E-02	*TAF12*, *MED18*, ***PRKDC***
FCR	PXR/RXR Activation	2.59E-02	*UGT1A1*, *FOXO3*
FCR	Granulocyte Adhesion and Diapedesis	2.62E-02	***SELP***, ***SELL***, *SELE*
FCR	Sphingomyelin Metabolism	2.96E-02	***SMPD2***
D110	Bile Acid Biosynthesis, Neutral Pathway	5.57E-08	***AKR1C3***, *AKR1C1/AKR1C2*, *AKR1C4*
D110	TR/RXR Activation	1.87E-05	*PFKP*, ***AKR1C3***, *AKR1C1/AKR1C2*
D110	Androgen Biosynthesis	3.32E-05	***AKR1C3***, *AKR1C4*
D110	Methylglyoxal Degradation III	4.38E-05	***AKR1C3***, *AKR1C1/AKR1C2*
D110	Retinoate Biosynthesis I	1.91E-04	***AKR1C3***, *AKR1C4*

Candidate genes selected for downstream validation are highlighted in bold

### SNP array validation

Four SNPs located near the QTLs for EBV FCR (rs80900450, rs319738340, rs340456509 and a novel SNP) in *PRKDC*, *SELL*, *NR2E1* and *OPRD1* respectively, and two SNPs mapped close to the QTL for EBV D110 (rs332368013 and rs81508945) in *MBD5* and *AKR1C3* respectively were confirmed to be polymorphic in target populations by sequencing. Subsequently, these six SNPs were genotyped in 436 Maxgro boars. Allelic frequencies and HWE are presented in Table 5. SNP in *MBD5* significantly departured from HWE (P-value < 0.05) indicating a slight deficiency of homozygotes in the studied population and SNP in *OPRD1* displayed minor allele frequency less than 5%. All SNPs, with the exception of the SNP in *MBD5* and *OPRD1* were tested for association with breeding values for FCR and D110 (Table 6). SNP rs80900450 and rs319738340 showed significant association with EBV FCR. Moreover, SNP rs340456509 was found significantly associated with both traits. The occurrence of the G allele was shown to be beneficial for both growth and feed efficiency. Significant additive effects of SNPs rs80900450, rs81508945 and rs340456509 were observed. In addition, SNP rs340456509 showed a dominant effect for EBV D110, however only one percent of the phenotypic variance was attributable to the dominant genetic variance.

**Table 5:** Observed and expected heterozygosity of the SNPs selected for validation.

Gene	SNP	Location (SSC 10.2)	Alleles	Variant	MAF	Observed heterozygosity	Expected heterozygosity	HWE^£^
*MBD5*	rs332368013	15:3,359,994	A/G	missense	0.300	0.471	0.420	0.014*
*NR2E1*	rs340456509	1:83,552,036	G/T	intron	0.211	0.359	0.334	0.155
*PRKDC*	rs80900450	4:87,256,301	C/T	missense	0.268	0.370	0.392	0.282
*SELL*	rs319738340	4:88,935,116	C/T	splice region	0.166	0.276	0.276	1
*AKR1C3*	rs81508945	10:72,102,793	G/C	missense	0.120	0.213	0.212	1
*OPRD1*	NOVEL	6:79,658,669	C/A	downstream	0.022	0.044	0.043	1

^£^p-value for test for departure from Hardy-Weinberg Equilibrium (HWE);

*Significant departure from HWE (p<0.05)

**Table 6:** Association of the five SNPs, located in selected functional genes mapped within 1Mb of the genome-wide significant markers, with breeding values for FCR and D110. Lower breeding values indicate higher feed efficiency.

SNP (gene)	Trait	P-value	Least squares means of EBVs per genotype			Additive effect		Dominant effect	
rs80900450 (*PRKDC*)			C/C n = 36	C/T n = 161	T/T n = 238	P-value	a^1^ (variance^2^)	P-value	d^1^ (variance^2^)
	EBV D110	0.085	-8.771 ±5.80	-8.416 ±5.78	-9.214 ±5.78				
	EBV FCR	**< .0001**	-0.036 ±0.15	-0.057 ±0.15	-0.084 ±0.15	**< .0001**	0.0377 (5%)	0.838	0.0026
rs319738340 (*SELL*)			C/C n = 303	C/T n = 120	T/T n = 12				
	EBV D110	0.366	-8.899 ±5.78	-8.389 ±5.78	-9.142 ±5.86				
	EBV FCR	**0.026**	-0.073 ±0.15	-0.049 ±0.15	-0.047 ±0.15	0.852	-0.0019	0.800	0.0048
rs340456509 (*NR2E1*)			G/G n = 265	T/G n = 156	T/T n = 14				
	EBV D110	**0.033**	-9.224 ±5.78	-8.327 ±5.78	-7.631 ±5.84	**< .0001**	-0.4849 (16%)	**0.044**	-0.2665 (1%)
	EBV FCR	**< .0001**	-0.085 ±0.15	-0.047 ±0.15	-0.021 ±0.15	**0.015**	-0.0230 (1%)	0.559	-0.0101
rs81508945 (*AKR1C3*)			C/C n = 6	C/G n = 93	G/G n = 337				
	EBV D110	0.195	-6.632 ±5.92	-8.561 ±5.79	-8.953 ±5.78				
	EBV FCR	0.468	-0.043 ±0.15	-0.058 ±0.15	-0.069 ±0.15				

Significant associations are in bold;

^1^Additive (a) and dominant (d) effect of an allelic substitution on a phenotype;

^2^ Phenotypic variance in percentage explained by SNP; Where delta was not significant, the alpha was reported from the first regression model.

## Discussion

In this study, a genome-wide association analysis was performed to elucidate the genetic architecture of feed conversion efficiency and growth rate in pigs. A number of candidate genes neighbouring the identified QTL regions were selected for downstream analysis. A further validation confirmed significant associations between these genes and EBV FCR / D110. The most prominent regions for EBV FCR were identified on SSC 1, 4, 6 and 15. For EBV D110, the most promising QTLs were detected on porcine chromosome 10 and 15. None of the identified QTL regions overlap for both traits. Alignment of the genetic and physical maps on the Sscrofa 10.2 genome assembly (PigQTLdb) enabled the identified QTLs from the present study to be compared with previously described QTL regions. A QTL from this study located at 78.3 to 80.5 Mb on SSC 6 coincided with a QTL for FCR in a European Wild Boar x Meishan cross mapped in the region of 127.3 cM (64.9 to 89 Mb, PigQTLdb) [18]. Additionally this QTL overlapped with a QTL for body weight detected at 78.3 to 78.7 Mb in Iberian x Landrace and Iberian x Meishan crosses [19]. This QTL has thus been independently discovered in different populations, which supports attributing it to biologically relevant common genetic variation [20]. QTL located at 86.7 to 89.1 Mb on SSC 4 found in this study was in a close proximity to QTL for FCR in a European Wild Boar x Pietrain cross mapped by Cepica et al. [21] at 75 cM (89.5 to 98.2 Mb, PigQTLdb). Another QTL on SSC 4 was detected at 20 cM (7.2 to 12.6 Mb, PigQTLdb) in a three-generation full-sib population, created by crossing Pietrain sires with Large White x Landrace x Leicoma dam line [22], which is very distant from the QTL identified in this study. These QTLs were detected by linkage analysis and therefore were mapped with very low resolution and cover large intervals. A genome-wide association study in a Danish Duroc population identified QTL for FCR located on SSC 4 at 63.8 to 64 Mb [6]. Another GWAS revealed QTL for FCR on SSC 4 at 4 to 5 Mb in a Duroc terminal sire population [8]. The remaining QTL regions for EBV FCR / D110 identified in this study on SSC 1, 10 and 15 did not colocalize closely to regions affecting FCR and growth rate found in the literature. Furthermore, Jiao et al. [8] mapped a QTL for daily feed intake in Duroc boars at 73.1 to 73.9 Mb, which is ~700 Kb from the QTL for EBV D110 detected in the present study. The very small number of overlapping QTL regions is in accordance with Gregersen et al. [23] who reported limited overlap of QTL for a particular trait between breeds. This might suggest that different QTLs regulate feed efficiency traits in the Maxgro boars compared to other breeds [4]. Moreover, the EBVs, which were used as response variable in the current study, are known to behave differently compared to raw phenotypes. EBVs have been reported to be more independent from environmental factors compared to raw phenotypes [20]. However, a recent evaluation of the direct use of EBVs for GWAS revealed issues of power, type I error and QTL effect sizes related to the incorporation of familial information in the estimation of EBV [16]. To account for these weaknesses linked to EBVs, the familial relationship (i.e. as genomic relationship matrix) was included in the statistical model as previously applied in other association analyses using EBVs [24–26]. The comparison of results obtained from different GWAS methods revealed that the used methodologies provide a further source for variation of results between different studies [27].

### Pathways and biological functions of genes mapped near the significant SNPs

Functional annotation revealed a number of pathways and biological processes significantly overrepresented among the positional candidate genes for EBV FCR and D110. Nearby genes to the significant markers for EBV FCR (*SELP*, *SELL*, *FGR*, *SELE*, *F5*, *FOXO3* and *OPRD1*) were identified to be involved in immune cell trafficking. Similarly, *THEMIS2*, *SELP*, *SELL*, *SELE*, *F5* and *PRKDC* were clustered in lymphoid tissue structure and development category. It is well documented that the activity of the immune system is linked to feed intake and therefore provide a relevant aspect for feed efficiency [28]. When immune response is activated, the available energy resources are shifted away from skeletal muscle accretion and prioritised to production of antibodies in order to fight the infection. This in turn might result in reduced rates of weight gain and feed conversion [29]. In addition, functional annotation of the positional candidate genes for EBV FCR to biological processes revealed a cluster of seventeen genes overrepresented in an organ morphology category (*CEBPD*, *SELL*, *ECEL1*, *NR2E1*, *SELE*, *CHRNG*, *WASF1*, *GPR3*, *EIF4E2*, *HTR2B*, *CHRND*, *SELP*, *KIFAP3*, *SEC63*, *SYTL1*, *PRKDC* and *FOXO3*). A study conducted by Njoku et al. [30] on Large White pigs revealed that visceral organ growth is stimulated by feed intake. Moreover, low RFI pigs have been associated with decreased visceral organ weight [31,32]. This is in agreement with Ferrell and Jenkins [33] postulating that a lower maintenance costs are associated with reduced visceral organ weight and decreased feed intake. A number of genes (*AKR1C3*, *MBD5*, *ACVR2A*, *AKR1C1/AKR1C2* and *AKR1C4*) located within 1Mb of the significant markers for EBV D110 were clustered in an endocrine system function and development category. Previous study identified smaller thyroid glands in low residual feed intake pigs [34]. Moreover, Gabarrou et al. [35] reported a decreased thyroid function in low RFI cockerels. Additionally, these genes belonging to Aldo-Keto Reductase family were significantly overrepresented in lipid metabolism and energy production. Lipid metabolism pathway as well as energy pathway were statistically associated with residual feed intake in muscle and adipose tissue of pigs [36–38].

### Candidate genes for feed conversion efficiency

Positional and functional genes located within 1 Mb of the GWAS SNPs significantly associated with breeding values for FCR/D110 were selected and examined. On SSC 4, SNP rs80900450 and rs319738340 in *PRKDC* and *SELL* respectively, were significantly associated with breeding value for FCR. ***PRKDC*** is a gene encoding the catalytic subunit of the DNA-dependent protein kinase (DNA-PK), which plays a part in DNA double stranded break repair [39]. *PRKDC* is involved in the signalling pathway responsible for the formation of fat from carbohydrates in the liver [40]. Wong et al. [40] conducted a study, in which they postulated that during fasting, inactivation of Fatty Acid Synthase (*FAS*) promoter occurs. However upon feeding, the *FAS* promoter becomes activated by *PRKDC* gene. In *PRKDC* deficient scid (severe combined immunodeficient) mice, feeding-induced transcriptional activation of the *FAS* gene and lipogenesis were impaired. As a result, reduced triglyceride level and decreased adipose tissue in *PRKDC* deficient scid mice were observed [40]. L-selectin (**SELL**) plays a role in lymphocyte trafficking to lymph nodes and Peyer’s patches, as well as targeting lymphocytes and neutrophils to an inflammation source [41]. The *SELL* encoded protein is a member of selectins belonging to a family of transmembrane glycoproteins and its role is to support adhesion of blood leucocytes to the vessel wall upon inflammatory and immunological response [42]. Significant reduction of L-selectin, which could affect the neutrophil’s ability to activate and travel to a source of inflammation, was observed in morbidly obese patients [43]. Yang et al. [44] proposed that L-selectin is responsible for mediating leukocyte homing to islets which would suggest it might be associated with autoimmune disease such as diabetes mellitus. Moreover, T668C SNP in *SELL* was associated with insulin-dependent diabetes mellitus [45]. Additionally, allele L206 of L-selectin gene was associated with inflammatory bowel disease [46].

**NR2E1** is a member of a ligand dependent transcriptional factors group, which controls a number of biological and disease related processes. *NR2E1* is abundantly expressed in the brain where it is involved in neurogenesis [47]. Christie et al. [48] and Kumar et al. [49] reported reduced neurogenesis in adult mice with *NR1E1* deletion. Moreover, the *NR2E1* knockout mice had reduced volume of olfactory bulb [49], a first central structure involved in processing of the olfactory information [50]. Interestingly, in the present study rs340456509 SNP in the *NR1E2* was significantly associated with breeding values for FCR and D110. Olfactory bulb plays an important part in regulating food intake as it is targeted by signals responsible for the regulation of energy balance [50], therefore it is hugely relevant for feed conversion efficiency.

***AKR1C3*** belongs to a large aldo and keto reductase enzyme family and is expressed in a wide variety of tissues including liver and adipose tissue. The protein encoded by this gene plays a role in conversion of active androgens, oestrogens and prostaglandins to their non-active metabolites [51]. *AKR1C3* has been associated with androgen inactivation induced adiposity, where large adipocytes had higher expression level compared to small adipocytes [52,53]. This finding was supported by a study conducted on obese patients having decreased *AKR1C3* expression upon diet induced weight loss [52]. Svensson et al. [52] also postulated that there might be a link between the *AKR1C3* gene and glucose intolerance. Moreover, White et al. [51] found an association between rs2211623 SNP and liver inflammation, which in turn might be related to insulin resistance. Nevertheless, in this study the selected SNP rs81508945 SNP in the *AKR1C3* was not found significantly associated with the breeding values for FCR or D110.

**MBD5** is a member of the methyl-CpG-binding domain (MBD) family of proteins. It is highly expressed in neurons [54] and is involved in mediating DNA methylation [55]. MBD5 also plays an essential role in the regulation of postnatal growth and glucose homeostasis [56]. A study conducted on *MBD5* knockout mice revealed severe growth retardation and persistent hypoglycemia, hypoinsulinemia, enhanced glucose intolerance and elevated insulin sensitivity. Moreover, mice lacking the *MBD5* gene exhibited significantly smaller body size and reduction of subcutaneous and perigonadal fat [56]. Nevertheless, in this study the selected SNP rs332368013 in the *MBD5* was out of HWE. The protein encoded by ***OPRD1***, a member of the opioid family of G-protein-coupled receptor, is broadly distributed in a number of brain areas involved in the regulation of energy homeostasis [57]. In particular, *OPRD1* is highly expressed in olfactory bulb and anterior olfactory nucleus [58]. *OPRD1* knockout mice fed with high energy diet were found to be resistant to weight gain and had lower fat mass. They also exhibited higher energy expenditure due to increased thermogenic activity in the brown adipose tissue [57]. Additionally, a number of SNPs within the *OPRD1* gene were significantly associated with anorexia nervosa [59,60]. Although it would be interesting to examine the role of *OPRD1* for feed efficiency and growth, the minor allele frequency of the identified novel SNP within this gene was lower than 5 percent and thus it was excluded from the further analysis.

## Conclusions

In summary, the present study demonstrated a number of chromosomal regions significantly associated with feed conversion efficiency and growth rate in the examined terminal pig sire line. Most of the regions were described for the first time, although some of them were located not far from previously reported QTLs. Validation of putative candidate genes from GWAS mapping near the significant SNPs confirmed a number of genes significantly associated with feed conversion efficiency and its related trait, days to 110 kg. Feed efficiency is a highly complex trait affected by a number of factors. This study suggests that the genetic predisposition of analysed traits is driven by lipogenesis, olfactory reception, and also immunological status. In depth characterisation of these genes to determine their molecular architecture and identify the causative mutations would be of benefit. Moreover, it would be useful to validate these SNPs in other commercial pig population regarding their effects on feed conversion efficiency and growth rate.

## Supporting information

S1 Table Chromosomal position and minor allele frequency (MAF) of markers significantly (-log10[p-value] ≥ 4.7) associated with breeding value of days to 110 kg (BV_D11) and breeding value of feed conversion ratio (BV_FCR) in a commercial pig population (n = 940). (PDF)

## Abbreviations

BLUP: best linear unbiased prediction
D110: days to 110 kg
EBV: estimated breeding value
FCE: feed conversion efficiency
FCR: feed conversion ratio
GWAS: genome-wide association study
MAF: minor allele frequency
QTL: quantitative trait locus
RFI: residual feed intake
SNP: single nucleotide polymorphism
SSC: Sus scrofa chromosome
