# Supplementary material for: Genome-wide association analysis and functional annotation of positional candidate genes for feed conversion efficiency and growth rate in pigs
Source: PLoS One. 2017 Jun 12;12(6):e0173482. doi: 10.1371/journal.pone.0173482 (PMC5467825; doi:10.1371/journal.pone.0173482)
Supplement: S1 Table — (PDF) [file pone.0173482.s001.pdf]

S1 Table. Chromosomal position and minor allele frequency (MAF) of markers significantly (-log10[p-value] ≥ 4.7) associated with breeding value of days to 110 kg (BV_D11) and breeding value of feed conversion ratio (BV_FCR) in a commercial pig population (n = 940).

Trait	Marker	Chromosome (*Sus scrofa* 10.2)	Position (bp)	Major allele	Minor allele	$-log_{10}$(P-value)	MAF
BV_D110	ALGA0000741	1	9984842	G	A	8.031	0.170
BV_D110	H3GA0002001	1	81518643	C	A	5.717	0.447
BV_D110	DRGA0001418	1	112480653	C	T	4.882	0.491
BV_D110	ALGA0005287	1	112505390	A	G	4.882	0.491
BV_D110	H3GA0002448	1	112726301	T	C	5.431	0.496
BV_D110	ASGA0005521	1	214059952	A	G	5.191	0.465
BV_D110	DIAS0004493	1	280835623	G	A	5.968	0.193
BV_D110	H3GA0004624	1	292182409	T	C	5.295	0.287
BV_D110	ALGA0009914	1	292235696	T	C	4.900	0.360
BV_D110	ASGA0013123	3	5827367	C	A	5.075	0.364
BV_D110	H3GA0008906	3	15634578	G	A	4.930	0.345
BV_D110	ASGA0090908	3	18873908	C	T	5.302	0.152
BV_D110	ALGA0102956	3	18876343	G	A	5.302	0.152
BV_D110	MARC0036115	3	72009115	C	T	6.076	0.343
BV_D110	MARC0050071	3	72016063	A	G	5.986	0.346
BV_D110	ASGA0015479	3	102763903	T	C	4.769	0.177
BV_D110	MARC0024866	3	138933442	G	A	5.253	0.036
BV_D110	ALGA0022593	4	6456384	A	G	5.077	0.079
BV_D110	ALGA0024359	4	33568631	T	C	4.861	0.358
BV_D110	ALGA0024373	4	33718639	G	A	4.861	0.358
BV_D110	ALGA0024372	4	33740194	A	G	4.861	0.358
BV_D110	MARC0017785	4	33903588	G	A	4.952	0.470
BV_D110	H3GA0012460	4	34124914	C	T	5.441	0.376
BV_D110	ASGA0019136	4	85824398	T	C	4.922	0.362
BV_D110	ISU10000732	4	111821142	G	A	5.162	0.069
BV_D110	ALGA0027450	4	112112443	T	C	5.360	0.065
BV_D110	MARC0052776	5	35527233	C	A	4.873	0.238
BV_D110	H3GA0017183	5	99245749	G	A	5.494	0.150
BV_D110	ASGA0084609	5	99603393	T	G	4.795	0.126
BV_D110	ALGA0108236	6	80473215	G	A	5.102	0.444
BV_D110	ALGA0035847	6	80577487	A	C	6.363	0.443
BV_D110	ALGA0049088	8	114685369	C	T	5.471	0.354
BV_D110	ASGA0091541	8	116042287	A	G	5.609	0.404
BV_D110	ALGA0049119	8	117393889	A	G	4.789	0.330
BV_D110	ASGA0044603	9	135580018	A	G	5.095	0.052
BV_D110	ASGA0099386	9	151674877	A	C	5.083	0.037
BV_D110	ASGA0085614	9	151691472	C	T	5.083	0.037
BV_D110	ASGA0103446	10	70669410	T	G	5.158	0.276
BV_D110	ASGA0087707	10	72261049	C	T	5.413	0.356
BV_D110	ALGA0060013	10	72375760	G	T	6.322	0.437
BV_D110	H3GA0030777	10	72766001	G	A	6.922	0.427
BV_D110	ALGA0119579	10	73404291	A	G	5.530	0.289
BV_D110	ASGA0049546	11	7107145	G	A	4.974	0.286
BV_D110	ALGA0060585	11	7134345	C	A	5.481	0.361
BV_D110	ALGA0061414	11	23577849	C	T	4.898	0.403
BV_D110	ASGA0103038	12	2690149	C	T	6.916	0.130
BV_D110	MARC0027759	12	57625866	C	T	5.406	0.448
BV_D110	DRGA0012390	13	57608490	T	C	5.741	0.214
BV_D110	DRGA0012397	13	58087990	A	G	5.368	0.246
BV_D110	DRGA0012399	13	58159422	G	A	5.689	0.209

S1 Table. Continued

Trait	Marker	Chromosome (*Sus scrofa* 10.2)	Position (bp)	Major allele	Minor allele	$-\log_{10}$(P-value)	MAF
BV_D110	M1GA0017545	13	59460430	G	A	5.679	0.252
BV_D110	ALGA0073466	13	199927172	T	C	5.353	0.058
BV_D110	ALGA0079898	14	99669710	C	T	5.390	0.294
BV_D110	ALGA0081112	14	121527590	C	T	5.450	0.441
BV_D110	DRGA0014640	14	136239246	T	G	6.592	0.102
BV_D110	MARC0036947	15	2640639	C	T	6.921	0.151
BV_D110	ALGA0115976	15	2798633	A	C	6.885	0.247
BV_D110	ALGA0113899	15	2835746	C	T	6.784	0.181
BV_D110	MARC0072361	15	2843921	T	C	7.753	0.141
BV_D110	ALGA0083417	15	3322649	G	A	8.146	0.144
BV_D110	DRGA0014782	15	3449792	T	C	5.424	0.322
BV_D110	MARC0034611	15	4129181	C	T	5.224	0.131
BV_D110	MARC0034674	15	4405843	C	T	5.247	0.397
BV_D110	MARC0033141	15	7988656	C	T	5.004	0.239
BV_D110	MARC0092322	15	98033621	G	C	5.244	0.064
BV_D110	ALGA0090172	16	35245008	G	A	5.057	0.256
BV_D110	SIRI0000298	16	35311791	A	G	5.057	0.256
BV_D110	ASGA0073002	16	35661077	T	G	5.028	0.209
BV_D110	INRA0052777	17	16899381	A	G	5.370	0.034
BV_D110	MARC0061919	17	16920977	G	A	4.902	0.031
BV_D110	ALGA0100254	X	138410450	C	T	5.384	0.040
BV_FCR	H3GA0002102	1	84686166	G	A	6.324	0.156
BV_FCR	ALGA0007869	1	239852452	T	C	5.942	0.133
BV_FCR	ALGA0004358	1	83904334	T	C	5.891	0.204
BV_FCR	ASGA0003509	1	84055266	A	G	5.891	0.204
BV_FCR	MARC0014911	1	83260076	G	T	5.859	0.207
BV_FCR	ASGA0003476	1	83283489	A	G	5.859	0.207
BV_FCR	DRGA0001159	1	83357328	A	G	5.744	0.235
BV_FCR	INRA0002912	1	83477121	T	C	5.699	0.236
BV_FCR	ASGA0003483	1	83498280	G	A	5.699	0.236
BV_FCR	ALGA0004354	1	83753095	T	G	5.699	0.236
BV_FCR	ASGA0003503	1	84007856	C	T	5.699	0.236
BV_FCR	ASGA0003506	1	84029992	A	G	5.676	0.210
BV_FCR	ASGA0003481	1	83444533	T	C	5.632	0.211
BV_FCR	H3GA0002049	1	83540558	T	C	5.632	0.211
BV_FCR	H3GA0002051	1	83812379	C	T	5.632	0.211
BV_FCR	MARC0091785	1	230057074	C	T	5.107	0.216
BV_FCR	MARC0100294	1	84107174	G	A	5.033	0.153
BV_FCR	ASGA0005720	1	242311874	T	C	4.992	0.114
BV_FCR	MARC0056239	1	242741853	C	T	4.992	0.112
BV_FCR	MARC0067813	1	242886911	C	T	4.992	0.112
BV_FCR	MARC0013705	1	103129617	C	T	4.887	0.399
BV_FCR	ALGA0000741	1	9984842	G	A	4.878	0.170
BV_FCR	MARC0025200	1	93730143	A	G	4.768	0.412
BV_FCR	ALGA0004441	1	84640944	C	T	4.759	0.122
BV_FCR	ALGA0010607	1	302880686	T	C	4.750	0.200
BV_FCR	ALGA0114773	2	152813354	A	C	5.708	0.482
BV_FCR	M1GA0004469	3	79317961	C	T	5.332	0.339
BV_FCR	ASGA0085168	3	62155304	C	G	5.006	0.429
BV_FCR	ASGA0089538	3	75159934	A	G	4.995	0.117
BV_FCR	H3GA0013204	4	88311790	G	A	7.961	0.416
BV_FCR	ALGA0026233	4	89118147	C	T	6.913	0.454
BV_FCR	ALGA0026230	4	89104182	A	G	6.841	0.463
BV_FCR	ALGA0024222	4	30060941	C	T	6.721	0.485
BV_FCR	M1GA0005738	4	15395576	C	T	6.693	0.053

S1 Table. Continued

Trait	Marker	Chromosome (*Sus scrofa* 10.2)	Position (bp)	Major allele	Minor allele	$-log_{10}$(P-value)	MAF
BV_FCR	ALGA0026204	4	87021547	C	A	6.469	0.461
BV_FCR	ALGA0023631	4	17248104	A	G	6.293	0.130
BV_FCR	MARC0000845	4	86747415	G	A	6.260	0.346
BV_FCR	MARC0006674	4	30077359	T	C	6.205	0.483
BV_FCR	ASGA0020471	4	88358606	G	A	5.728	0.473
BV_FCR	ASGA0020473	4	88385240	T	C	5.728	0.473
BV_FCR	ALGA0025816	4	78608608	T	C	5.722	0.444
BV_FCR	MARC0021448	4	78622798	T	C	5.722	0.444
BV_FCR	ASGA0020148	4	78641029	C	T	5.722	0.444
BV_FCR	ALGA0026374	4	93378763	T	C	5.692	0.215
BV_FCR	ASGA0019062	4	27684348	A	C	5.545	0.478
BV_FCR	M1GA0005974	4	82154248	C	T	5.383	0.467
BV_FCR	ASGA0093554	4	16069475	G	A	5.331	0.370
BV_FCR	ASGA0090485	4	16070229	A	G	5.331	0.370
BV_FCR	ALGA0116077	4	86261296	G	A	5.294	0.310
BV_FCR	ASGA0020466	4	87889676	T	G	5.271	0.477
BV_FCR	MARC0009527	4	88437516	A	C	5.120	0.254
BV_FCR	CASI0007993	4	88762691	A	G	5.120	0.254
BV_FCR	ASGA0020454	4	87007876	G	A	5.094	0.224
BV_FCR	ASGA0020384	4	83846382	A	G	4.959	0.465
BV_FCR	MARC0083264	4	89273187	G	A	4.916	0.357
BV_FCR	ALGA0108541	4	133102015	A	C	4.910	0.123
BV_FCR	ALGA0024155	4	28144078	G	A	4.772	0.221
BV_FCR	ASGA0021673	4	114804404	T	C	4.745	0.451
BV_FCR	ASGA0018763	4	18153487	T	C	4.735	0.485
BV_FCR	M1GA0008166	5	106059180	G	T	7.521	0.143
BV_FCR	ASGA0026626	5	90128642	T	C	6.800	0.366
BV_FCR	ASGA0027134	5	105954521	G	A	6.618	0.482
BV_FCR	DRGA0006468	5	108606349	A	G	5.419	0.078
BV_FCR	DRGA0006480	5	108885783	G	A	5.419	0.078
BV_FCR	DRGA0006449	5	109415562	A	G	5.419	0.078
BV_FCR	ASGA0024351	5	9942508	T	C	4.769	0.498
BV_FCR	MARC0031617	6	127646479	A	G	6.577	0.483
BV_FCR	ASGA0028724	6	78297229	A	G	6.443	0.351
BV_FCR	ALGA0035847	6	80577487	A	C	6.434	0.443
BV_FCR	ALGA0108236	6	80473215	G	A	5.939	0.444
BV_FCR	MARC0083933	6	78053471	G	A	5.820	0.423
BV_FCR	ASGA0095713	6	7643426	T	C	5.802	0.269
BV_FCR	ASGA0028717	6	78093507	T	C	5.642	0.432
BV_FCR	M1GA0025424	6	149384762	G	A	5.375	0.247
BV_FCR	ASGA0099240	6	78503421	C	T	5.220	0.426
BV_FCR	MARC0018089	6	79884591	C	T	5.136	0.285
BV_FCR	ALGA0037010	6	130083282	A	G	4.793	0.246
BV_FCR	ALGA0124052	6	80979535	G	A	4.717	0.094
BV_FCR	ALGA0045445	7	125434386	A	G	6.827	0.065
BV_FCR	ALGA0043503	7	95646427	A	G	5.807	0.185
BV_FCR	ASGA0037781	8	10172634	G	A	6.342	0.473
BV_FCR	ASGA0041689	9	13309995	A	C	6.614	0.466
BV_FCR	ALGA0118156	9	7629819	G	A	6.035	0.347
BV_FCR	ALGA0055001	9	133403289	A	G	5.845	0.285
BV_FCR	H3GA0028548	9	145215254	A	G	5.549	0.223
BV_FCR	H3GA0055365	9	7629459	A	G	5.373	0.377
BV_FCR	ALGA0122939	9	132566139	A	G	5.099	0.371
BV_FCR	ALGA0060013	10	72375760	G	T	5.156	0.437
BV_FCR	ALGA0057031	10	12263111	C	T	5.080	0.112

S1 Table. Continued

Trait	Marker	Chromosome (*Sus scrofa* 10.2)	Position (bp)	Major allele	Minor allele	-log$_{10}$(P-value)	MAF
BV_FCR	H3GA0029253	10	13119118	C	T	4.819	0.313
BV_FCR	ALGA0061169	11	18669144	A	C	5.603	0.324
BV_FCR	MARC0049886	11	23923832	G	A	5.600	0.383
BV_FCR	DRGA0010905	11	15846590	C	A	4.713	0.444
BV_FCR	MARC0021670	12	37005248	A	G	5.894	0.050
BV_FCR	H3GA0034902	12	58064000	A	G	5.023	0.372
BV_FCR	ASGA0101792	12	39587325	G	T	4.810	0.326
BV_FCR	DRGA0011805	12	51938171	G	T	4.716	0.161
BV_FCR	M1GA0025611	12	52131264	C	T	4.716	0.161
BV_FCR	ALGA0073977	13	213948108	C	T	5.857	0.116
BV_FCR	MARC0029581	13	15811761	A	G	5.601	0.071
BV_FCR	H3GA0041606	14	104899055	T	C	6.675	0.098
BV_FCR	ASGA0061606	14	14998570	T	C	5.332	0.104
BV_FCR	DRGA0013766	14	30612689	C	T	4.717	0.182
BV_FCR	MARC0015113	15	146404317	A	G	7.400	0.063
BV_FCR	MARC0054210	15	61968193	G	A	6.461	0.081
BV_FCR	ALGA0119312	15	149350761	G	A	6.271	0.184
BV_FCR	ALGA0110207	15	149318714	A	G	5.899	0.195
BV_FCR	INRA0058294	15	151595855	T	C	5.694	0.356
BV_FCR	MARC0082913	15	49748365	G	T	5.028	0.071
BV_FCR	MARC0034674	15	4405843	C	T	4.793	0.397
BV_FCR	DRGA0016308	16	72717040	G	A	6.170	0.080
BV_FCR	MARC0015715	16	81970866	C	T	5.035	0.193
BV_FCR	ASGA0098951	16	74560093	T	C	4.765	0.267
BV_FCR	ASGA0076498	17	37552167	G	A	5.308	0.242
BV_FCR	ASGA0103482	17	11648411	T	C	5.226	0.462
BV_FCR	ALGA0114599	17	50466682	T	C	5.159	0.107
BV_FCR	ASGA0085139	17	50468152	T	G	5.159	0.107
BV_FCR	M1GA0023780	X	125569642	G	A	8.267	0.111
BV_FCR	ALGA0111101	X	2581887	T	C	6.708	0.424
BV_FCR	ALGA0106978	X	2556821	T	C	6.463	0.396
BV_FCR	ASGA0081152	X	45539525	T	C	6.001	0.469
BV_FCR	DRGA0017254	X	45561094	C	T	6.001	0.469
BV_FCR	H3GA0051784	X	45972443	C	T	5.839	0.415
BV_FCR	ALGA0099925	X	110666451	G	A	5.822	0.369
BV_FCR	ASGA0081451	X	125698970	G	A	5.499	0.126
BV_FCR	ALGA0099728	X	45914845	G	A	5.473	0.207
BV_FCR	ALGA0099288	X	8692219	C	T	5.176	0.469
BV_FCR	MARC0056935	X	125199118	G	A	5.115	0.134
BV_FCR	M1GA0023703	X	42908366	T	C	5.115	0.216
BV_FCR	ASGA0080749	X	8718698	C	T	5.087	0.447
BV_FCR	ALGA0100103	X	125294839	C	T	4.902	0.134
BV_FCR	ALGA0099572	X	37678424	A	G	4.766	0.395
